# Antibodies to *Aedes aegypti* D7L salivary proteins as a new serological tool to estimate human exposure to *Aedes* mosquitoes

**DOI:** 10.3389/fimmu.2024.1368066

**Published:** 2024-05-01

**Authors:** Sophana Chea, Laura Willen, Sreynik Nhek, Piseth Ly, Kristina Tang, James Oristian, Roberto Salas-Carrillo, Aiyana Ponce, Paola Carolina Valenzuela Leon, Dara Kong, Sokna Ly, Ratanak Sath, Chanthap Lon, Rithea Leang, Rekol Huy, Christina Yek, Jesus G. Valenzuela, Eric Calvo, Jessica E. Manning, Fabiano Oliveira

**Affiliations:** ^1^ International Center of Excellence in Research, National Institute of Allergy and Infectious Diseases, Phnom Penh, Cambodia; ^2^ Laboratory of Malaria and Vector Research, National Institute of Allergy and Infectious Diseases, National Institutes of Health, Bethesda, MD, United States; ^3^ National Center for Parasitology, Entomology, and Malaria Control, Ministry of Health, Phnom Penh, Cambodia; ^4^ National Dengue Control Program, Ministry of Health, Phnom Penh, Cambodia

**Keywords:** *Aedes*, exposure marker, ELISA, dengue, Cambodia, mosquito saliva

## Abstract

**Introduction:**

*Aedes spp.* are the most prolific mosquito vectors in the world. Found on every continent, they can effectively transmit various arboviruses, including the dengue virus which continues to cause outbreaks worldwide and is spreading into previously non-endemic areas. The lack of widely available dengue vaccines accentuates the importance of targeted vector control strategies to reduce the dengue burden. High-throughput tools to estimate human-mosquito contact and evaluate vector control interventions are lacking. We propose a novel serological tool that allows rapid screening of human cohorts for exposure to potentially infectious mosquitoes.

**Methods:**

We tested 563 serum samples from a longitudinal pediatric cohort study previously conducted in Cambodia. Children enrolled in the study were dengue-naive at baseline and were followed biannually for dengue incidence for two years. We used Western blotting and enzyme-linked immunosorbent assays to identify immunogenic *Aedes aegypti* salivary proteins and measure total anti-*Ae. aegypti* IgG.

**Results:**

We found a correlation (rs=0.86) between IgG responses against AeD7L1 and AeD7L2 recombinant proteins and those to whole salivary gland homogenate. We observed seasonal fluctuations of AeD7L1+2 IgG responses and no cross-reactivity with *Culex quinquefasciatus* and *Anopheles dirus* mosquitoes. The baseline median AeD7L1+2 IgG responses for young children were higher in those who developed asymptomatic versus symptomatic dengue.

**Discussion:**

The IgG response against AeD7L1+2 recombinant proteins is a highly sensitive and *Aedes* specific marker of human exposure to *Aedes* bites that can facilitate standardization of future serosurveys and epidemiological studies by its ability to provide a robust estimation of human-mosquito contact in a high-throughput fashion.

## 1 Introduction

Dengue is the most prevalent arboviral disease worldwide, with the greatest burden in tropical and sub-tropical regions (1, 2). Half the world’s population is at risk of contracting dengue, including 1.3 billion people living in 10 dengue-endemic countries of the Southeast Asian region (3). From 2015 to 2019, dengue cases in the region increased by 46%, culminating in a major epidemic in 2019 that led to the highest number of annual global cases (>5.2 million) ever to be reported in the same year (4).

Currently, existing vaccines remain selectively available; thus, prevention and control of dengue heavily rely on effective vector control measures targeted toward *Aedes* mosquitoes. The efficacy of these vector control methods is currently evaluated using expensive and labor-intensive insect trapping that may not reflect true human exposure to mosquito bites. An alternative approach exploits the human humoral immune responses against the vector’s salivary components that are injected during a bloodmeal. Salivary proteins of several hematophagous arthropods were previously identified and shown to facilitate blood feeding by limiting the host’s vasoconstriction, inhibiting coagulation processes, and suppressing pain receptors (5). Saliva of *Aedes aegypti* female mosquitoes contains approximately 100 abundant salivary proteins (6, 7) that are implicated in enhanced viral dissemination and increased pathogenesis of viral infections (8).

IgG responses against whole salivary gland homogenate (SGH) of *Aedes* mosquitoes correlate with the intensity of mosquito bite exposure (9) and were previously proposed as a marker of human exposure to *Aedes* mosquitoes (10–12). Since then, researchers established a repertoire of promising recombinant salivary antigens to bypass the potential cross-reactivity of SGH with other vector species and to facilitate the use of biomarkers of *Aedes* exposure in large-scale cohort studies (13), with the Nterm-34kDa peptide biomarker being the most studied (13, 14). Interestingly, none of these studies assessed how well the antibody (Ab) responses against *Ae. aegypti* recombinant proteins correlate with those against whole saliva or SGH, the reference standard for *Aedes* exposure. Ab responses against whole SGH and several recombinant *Ae. aegypti* salivary proteins have also been implicated in associations with disease outcomes (15, 16). IgG reactivity to *Ae. aegypti* salivary proteins was higher in DENV-positive patients than in uninfected patients (9, 15, 17). Conversely, Ab responses against the *Ae. aegypti* D7 proteins, involved in the scavenging of biogenic amines and cysteinyl leukotrienes and, thus, potentially involved in preventing the host’s inflammatory response, were higher in DENV-positive and febrile patients as opposed to non-febrile patients (17). In the present study, we identified salivary biomarkers of *Ae. aegypti* exposure that can replace the use of mosquito saliva or SGH and subsequently investigated if a relationship exists between these surrogate biomarkers of exposure and clinical outcomes in dengue-infected patients in a longitudinal pediatric cohort in Cambodia (18).

## 2 Material and methods

### 2.1 Study population and sample selection

The samples selected for the present study originate from the PAGODAS longitudinal cohort study conducted in Cambodia, of which study protocol and results are reported elsewhere (18, 19). In brief, children enrolled in the study (2–9 years old) were followed up every 6 months for a period of 3 years to assess their immune status against dengue and *Ae. aegypti* saliva. Children were considered dengue naïve at the study baseline if they were negative on the PanBio Dengue Indirect IgG enzyme-linked immunosorbent assay (ELISA) or, when ELISA positive, they were negative for the presence of DENV-specific neutralizing Abs (n = 563). During the follow-up period, 41 children developed symptomatic dengue as confirmed by RT-PCR for DENV 1–4. Children who seroconverted *via* pan-DENV ELISA screening with a corresponding increase in DENV 1–4 neutralization Ab titers over the threshold value of 1:40 at one of the semiannual visits and who had not reported fever-like symptoms during active febrile surveillance were considered to have had asymptomatic dengue (n = 173).

For the present study, a random sample of baseline-dengue naïve children, stratified by quartiles of anti-SGH Ab response levels (i.e., high, medium, or low), were selected for the initial immunogenicity screening of *Ae. aegypti* salivary proteins by Western blotting (n = 102). Ab responses against five identified salivary antigens and combinations thereof were then measured in a random subset of samples collected at all seven visits from 18 children in the full cohort who were either dengue naïve or dengue immune at baseline (n = 126) to investigate correlations with the Ab response against whole SGH and to evaluate the seasonality of these Ab responses. Associations between anti-salivary protein Abs and disease outcomes (i.e., no dengue infection, symptomatic disease, or asymptomatic disease) were assessed, and correlations were confirmed using all baseline-dengue naïve samples (n = 563). Negative controls were selected from the National Institutes of Health (NIH) Clinical Center (CC) blood donor samples that were non-reactive against *Ae. aegypti* saliva (n = 10); positive controls were selected from human serum samples with high reactivity against *Ae. aegypti* SGH, as defined by ELISA, and stored at the International Centers for Excellence in Research laboratory.

### 2.2 Ethics

The study protocol was approved by the institutional review boards (IRBs) at the U.S. National Institutes of Health and the National Ethics Committee on Human Research (NECHR) in Cambodia. The parents or guardians of all pediatric participants provided signed informed consent to participate in the study (ClinicalTrials.gov identifier: NCT03534245). Sera from healthy U.S. blood donors were collected after participants provided signed informed consent in the National Institutes of Health Clinical Center IRB-approved protocol entitled *Collection and Distribution of Blood Components from Healthy Donors for In Vitro Research Use* (ClinicalTrials.gov identifier: NCT00001846).

### 2.3 Mosquito rearing, dissection, and salivary gland homogenization

*Ae. aegypti* (Kampong Speu strain, Cambodia and Liverpool strain, Rockville, MD, USA), *Aedes albopictus* (Phnom Penh strain, Cambodia), *Culex quinquefasciatus* (Phnom Penh strain, Cambodia), and *Anopheles dirus* (Mondulkiri strain, Cambodia) mosquitoes were reared in a controlled environment insectary at the National Center for Parasitology, Entomology and Malaria Control (Cambodia) under a 12-hour light–dark cycle, at 26°C, and 75% relative humidity. *Ae. aegypti* Rockefeller strain was reared under the same conditions at the Laboratory of Malaria and Vector Research (National Institutes of Health, USA). Larvae were fed with fish food (Tetra, Blacksburg, VA, USA) every 2 days until pupal development, while adults were offered 10% sucrose solution, *ad libitum*. Salivary glands from females 7–10 days post-eclosion were dissected under a stereomicroscope at one gland per microliter of sterile 1× phosphate-buffered saline (PBS), pH 7.4 (Thermo Fisher Scientific, Waltham, MA, USA) and stored at −80°C. Prior to use, salivary glands were disrupted through ultrasonication, and the supernatant was collected after the removal of insoluble debris by centrifugation at 14,000 rpm for 5 minutes at 4°C.

### 2.4 Production of recombinant *Ae. aegypti* salivary proteins

The human cell line HEK293 was used to recombinantly express 18 *Ae. aegypti* recombinant proteins, as described previously (20). After identification of the most immunogenic proteins, the production of AeApyrase, AeD7L1, AeD7L2, NIH-23, and NIH-27 was scaled up, and they were codon-optimized for mammalian expression, synthesized with a carboxyl-terminal HIS-tag (GenScript, Piscataway, NJ, USA), and cloned into pHEK293 Ultra Expression Vectors (Takara, Mountain View, CA, USA). The SAIC Advanced Research Facility (Frederick, MD, USA) then transfected the EXPI293F human cells and recombinantly expressed the proteins. Proteins were purified from the cell culture supernatant using AmMag Ni Magnetic beads (GenScript) and the AmMag Wand D12 (GenScript), following the manufacturer’s instructions. Protein dialysis was performed overnight with sterile PBS (Lonza, Basel, Switzerland) using 10K MWCO Slide-A-Lyzer Dialysis Cassettes (Thermo Fisher Scientific). The BCA Protein Assay Kit (Pierce) was used to quantify the protein concentration, after which sodium dodecyl sulfate–polyacrylamide gel electrophoresis (SDS-PAGE) was performed using NuPAGE Novex 4%–12% Bis-Tris gradient protein gels (Thermo Fisher Scientific) to assess the quality of the purified proteins. Proteins were visualized using Coomassie G-250 (SimplyBlue SafeStain, Invitrogen, Carlsbad, CA, USA) using the eStain protein staining device (GenScript), following the manufacturer’s instructions.

### 2.5 Enzyme-linked immunosorbent assay

Total anti-*Ae. aegypti* SGH IgG was measured by performing ELISAs as adapted from our previously described protocol (18). In brief, 96-well Immulon plates were coated with either *Ae. aegypti* SGH (2 µg/mL, Kampong Speu strain) or our *Ae. aegypti* recombinant salivary proteins (5 µg/mL, per protein for combined assays) diluted in carbonate–bicarbonate buffer (pH 9.6) and incubated overnight at 4°C. Plates were blocked with 200 μL of Tris-buffered saline with Tween-20/bovine serum albumin (TBST/BSA) 4% for 1 hour at room temperature (RT) and incubated with sera (diluted 1:200) for 2 hours at RT. Plates were washed three times with TBST. Secondary anti-human IgG Abs conjugated to alkaline phosphatase were added at a concentration of 1:10,000, diluted in TBST/BSA 4% (A1543, MilliporeSigma, Burlington, MA, USA), and incubated for 1 hour at RT. After six washes, the Alkaline Phosphatase Yellow (pNPP) substrate was added, and absorbance was read at 450 nm within 30 minutes. Serum samples were tested in duplicate and retested if the coefficient of variation was above 20%. The optical density (OD) value of the blank control, included in each plate, was subtracted from the raw sample OD values. The same set of positive and negative control samples was included in each plate (per antigen) to allow for inter-plate standardization [i.e., OD sample/(OD positive control − OD negative control)].

### 2.6 Western blotting

Western blotting experiments were performed to identify the most immunogenic *Ae. aegypti* salivary proteins. In brief, 50 µg of *Ae. aegypti* SGH (Rockefeller strain) or 5 µg of recombinant proteins was denatured at 70°C for 10 minutes under non-reducing conditions and loaded into one 2D well (NuPAGE™ 4%–12% Bis-Tris Gel, Thermo Fisher Scientific) or in each lane of a 10-well gel (Bolt™ 4%–12% Bis-Tris Plus, Thermo Fisher Scientific), respectively. Proteins were separated by SDS-PAGE at 200 V for 40 minutes. The proteins were transferred to a 0.45 µm nitrocellulose membrane (Thermo Fisher Scientific) and blocked with 5% non-fat dried milk dissolved in Tris-buffered saline with 0.05% Tween 20 (TBST–5% milk) (Sigma, St. Louis, MO, USA) at 4°C overnight. The next day, the membrane was incubated with serum samples (diluted 1:200) in TBST–5% milk. Samples were either individually loaded in the mini-protean II multiscreen apparatus (Bio-Rad, Hercules, CA, USA) (i.e., for the initial immunogenicity screening of whole SGH) or pooled and transferred to hybridization bags (Kapak SealPAK, ProAmpac, Cincinnati, OH, USA) (i.e., to verify the immunogenicity of the five identified proteins). After a 2-hour incubation at RT, the membranes were incubated for 1 hour at RT with secondary alkaline phosphatase-conjugated goat anti-human IgG heavy and light chains (IgG H+L; diluted at 1:10,000) (Sigma). Between each phase, membranes were washed three times for 5 minutes with TBST. The chromogenic reaction was initiated with Alkaline Phosphatase Western Blue^®^ Stabilized Substrate (Promega, Madison, WI, USA) and stopped with distilled water after approximately 10 to 15 minutes. Images were captured using a KwikQuant Digital Western Blot Detection System (Kindle Biosciences, Greenwich, CT, USA). Kindle Biosciences’ KwikQuant Image Analyzer software was used to analyze images and identify reactive protein bands. For the Western blotting inhibition assay, all *Aedes*-specific antibodies were first quenched by incubating the pooled sera with 5 μg of both recombinant AeD7L1 and AeD7L2 in hybridization bags overnight at RT prior to incubation with the membrane.

### 2.7 Phylogenetic analysis

The sequences of the salivary D7 long-form proteins from *Ae. aegypti*, *Ae. albopictus*, and *Cx. quinquefasciatus* were aligned after the removal of their predicted signal peptide sequence (21). Multiple sequence alignment and identity/similarity matrices were constructed in Geneious v2022.1.1 using the MUSCLE algorithm (22) and the PAM200 scoring matrix. Homologs of AeD7L1 and AeD7L2 were identified in the Non-Redundant (NR) protein database and the Transcriptome Shotgun Assembly (TSA) database using the Basic Local Alignment Search Tool (23). Mosquitoes with the most homologous sequences to *Ae. aegypti* (based on their E-value) were selected. The unrooted evolutionary tree was then constructed using PAUP 4.0 (24) in Geneious v2022.1.1, following the maximum likelihood procedure of the WAG amino acid substitution matrix model. The partial deletion option was applied for gaps/missing data. The reliability of the trees was tested by the bootstrap method (n = 1,000).

### 2.8 Statistical analysis

The normality of OD values was assessed by visual inspection of their distribution and formally tested using the Shapiro–Wilk test. A Kruskal–Wallis rank test was used to test for differences among >2 groups (for non-parametric inferences), with a post-hoc Dunn’s test. A Friedman’s rank test with a post-hoc Conover–Iman test was used to test for differences among more than two paired groups. Multiple comparisons were adjusted using the Benjamini–Hochberg procedure. Kendall’s W was used as a measure of Friedman’s test effect size. Changes in immune responses with age and disease outcomes were tested by fitting generalized linear models (GLMs) to the Ab values, where a gamma distribution and log-link function gave the best fit by log-likelihood goodness-of-fit statistics. An age × disease outcome interaction term was included in the model, with age treated as a mean-centered continuous variable. Correlations between anti-SGH and anti-recombinant protein IgG responses were estimated by Pearson’s method to investigate a linear relationship between both responses (r) and by Spearman’s rank method to assess monotonic relationships (r_s_). Cox proportional hazards (PH) models were used to model time to any dengue infection (in days), comparing those with high *vs.* low Ab responses at baseline and controlling for sex, age, insecticide use, larvicide use, bed net use, mosquito coil use, school attendance, socio-economic status, and number of toilets around the house. To fit the Cox PH models, Ab responses were categorized as high or low based on a cutoff established using tertiles of Ab responses: “high” is defined as the middle and highest tertiles and “low” as the lowest tertile. All statistical analyses were performed in R Statistical Software (25), and graphical representations were created using the “ggplot2” and “corrplot” packages in R (26, 27).

## 3 Results

### 3.1 Identification of immunogenic *Ae. aegypti* salivary proteins in a Cambodian pediatric population

**Figure 1:**
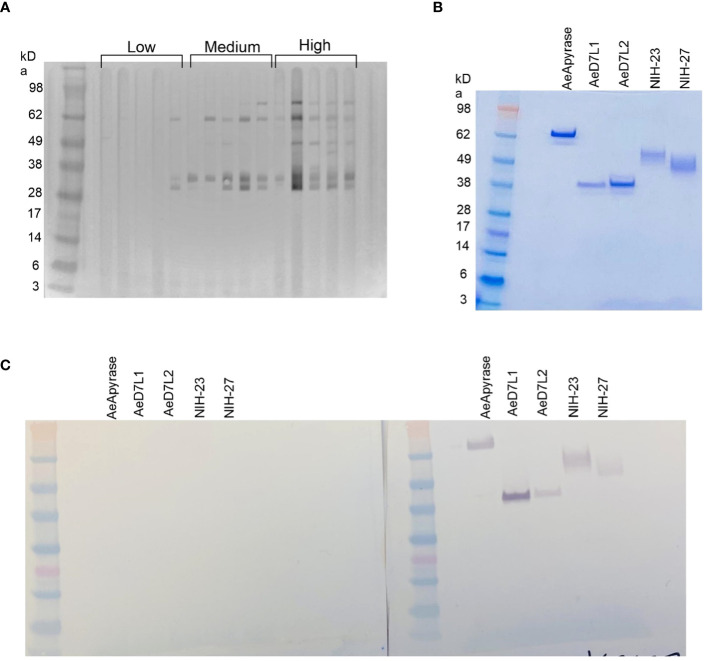
Sera from children living in dengue-endemic areas in Kampong Speu Province, Cambodia, recognize native salivary gland homogenate (SGH) from *Aedes aegypti*. **(A)** Western blotting displaying reactivity of Cambodian serum samples organized by increasing *Ae. aegypti* SGH ELISA optical density (OD) values, classified as “low” when OD < 0.162 (lower than the first quartile), “high” when OD > 0.4475 (higher than the third quartile), and “medium” if the OD fell in between the first and third quartiles. **(B)** Coomassie blue stained sodium dodecyl sulfate–polyacrylamide gel electrophoresis (SDS-PAGE) of the five selected recombinant proteins (AeApyrase, AeD7L1, AeD7L2, NIH-23, and NIH-27). **(C)** Western blotting with AeApyrase, AeD7L1, AeD7L2, NIH-23, and NIH-27 against a pool of negative (left) and positive (right) sera.

We used Western blotting to measure the recognition of *Ae. aegypti* salivary proteins by IgG present in the serum of our study population. We observed serum reactivity in the *Ae. aegypti* SGH molecular weight range of 28 kDa to 98 kDa (**Figure 1A**; **Supplementary Figure S1**). Cambodian serum reactivity increased on par with anti-SGH ELISA OD values (**Figure 1A**; **Supplementary Figure S1**). Using a digital detection system, we identified reactive protein bands with approximate molecular weights of 80 kDa, 62 kDa, 35 kDa, and 30 kDa, accounting for 72%, 88%, 85%, and 82% of the total seropositivity, respectively. We next produced and screened 18 *Ae. aegypti* recombinant salivary proteins by Western blotting using a pool of *Ae. aegypti* SGH reactive sera from our cohort (n = 10) (**Supplementary Figure S2**). We identified five of the 18 *Ae. aegypti* recombinant salivary proteins as reactive, corresponding to AeApyrase, AeD7L1, AeD7L2, NIH-23, and NIH-27 (**Table 1**; **Supplementary Figure S2**). We scaled up the production of these five *Ae. aegypti* salivary recombinant proteins, verified their purity by Coomassie G-250 staining (**Figure 1B**), and tested their reactivity to the pooled sera (**Figure 1C**). Importantly, sera from healthy U.S. blood donors who were non-reactive on an *Ae. aegypti* SGH ELISA also did not react to any of the tested recombinant proteins (**Figure 1C**).

**Table 1:** Name, molecular weight, accession number, serum reactivity, and function of the 18 recombinant proteins used for the immunoreactivity screening against *Aedes aegypti* salivary gland homogenate.

	Protein name	Molecular weight (kDa)	Accession number	Serum reactivity	Annotation
1	NIH435-36	20.05	ABF18174	No	Putative 14.5-kDa salivary protein (28)
2	NIH435-8	20.6	AAL76029	No	Putative C-type lectin (29)
3	NIH435-4	21	ABF18046	No	Proline-rich salivary secreted peptide (28)
4	NIH435-29	21.16	AAL76036	No	Putative 18.2-kDa secreted protein (29)
5	AeD7S	21.25	ABF18082	No	Putative odorant binding protein (28)
6	Aegyptin	30	CAX36783	No	Binds collagen and inhibits platelet aggregation and adhesion (30)
7	NIH435-32	36.4	ABF18025	No	Angiopoietin-like protein variant and fibrinogen-related domains (FReDs) (28)
8	NIH435-27	36.7	AAL76019	Yes	Putative sticky protein, angiopoietin-related protein, and fibrinogen-related domains (29)
9	AeD7L1	37	AAL16049	Yes	Binds biogenic amines and eicosanoids (31)
10	AeD7L2	37	AAA29347	Yes	Binds biogenic amines and eicosanoids (31)
11	NIH435-31	38.72	ABF18017	No	Putative 34-kDa family secreted salivary protein (28)
12	NIH435-24	39.13	XP_001657055	No	Uncharacterized protein (32)
13	NIH435-16	20.05	XP_001651977	No	Probable uridine nucleosidase 1 and nucleoside hydrolases (32)
14	NIH435-23	20.6	XP_001656512	Yes	Serine protease inhibitors (Serpin) family and clotting inhibitor (33, 34)
15	NIH435-19	21	EAT46066	No	Serine protease inhibitors (Serpin) family and clotting inhibitor (33)
16	NIH435-6	21.16	ABF18028	No	Salivary anti FXa serpin (28)
17	NIH435-21	21.25	XP_001663083	No	Cysteine-rich secretory proteins, antigen 5, and pathogenesis-related 1 proteins (32)
18	AeApyrase	61.36	AAC37218.1	Yes	Inhibiting ADP-dependent platelet aggregation (35)

### 3.2 The combined antibody responses against *Ae. aegypti* D7L1 and D7L2 salivary proteins is a valid surrogate for antibodies against *Ae. aegypti* SGH

**Figure 2:**
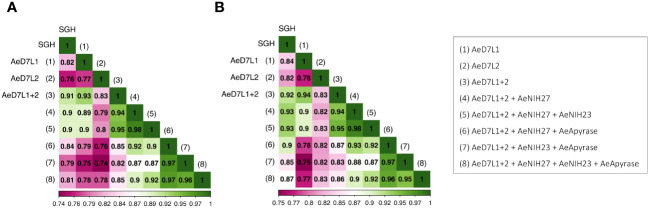
Heat map showing Pearson’s **(A)** and Spearman’s **(B)** correlation coefficients (r and r_s_, respectively) between antibody responses against salivary gland homogenate (SGH) and against AeD7L1+2, AeD7L1, or AeD7L2, alone or when combined with AeApyrase, AeNIH-27, or AeNIH-23.

We tested a random subset of 18 Cambodian children sampled at seven consecutive visits during the dry and wet seasons from 2018 to 2021 on an ELISA for Abs against the selected recombinant proteins or SGH (**Figure 2A**). We achieved a high correlation between SGH and AeD7L1+2 (r = 0.91, r_s_ = 0.92), AeD7L1 (r = 0.82, r_s_ = 0.84), and AeD7L2 (r = 0.76, r_s_ = 0.82), alone or when combined with AeApyrase, AeNIH-27, or AeNIH-23 (**Figure 2**). We observed no significant correlation between Abs against SGH and a negative control antigen (BSA) [r = 0.17 (*p* = 0.05), r_s_ = 0.15 (*p* = 0.1)]. We selected the combination of AeD7L1+2 as the optimal and most parsimonious candidate for further analyses. All *p*-values and confidence intervals are displayed in **Supplementary Table 1**.

### 3.3 Antibody responses show a seasonal fluctuation that is most pronounced for AeD7L1 and AeD7L1+2

**Figure 3:**
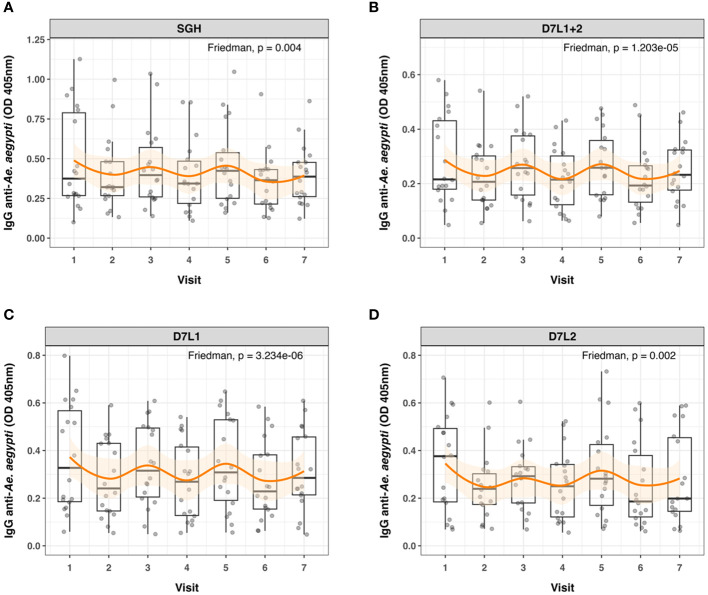
Seasonal variation in anti-*Aedes aegypti* IgG levels. Dot and box plots of standardized ELISA optical density (OD) values of 18 individuals tested at seven different time points (Visits 1 to 7) for IgG against **(A)** salivary gland homogenate (SGH), **(B)** AeD7L1+2, **(C)** AeD7L1, and **(D)** AeD7L2. Visits 1, 3, 5, and 7 took place in wet seasons from 2018 to 2021; the other visits took place during the dry seasons. The solid black horizontal line within the box plots is the median; the lower and upper borders are, respectively, the first and third quartiles; the vertical bars indicate the minimum and maximum values. The orange smoothed fitted curves represent a loess regression (span α = 0.45) with 95% confidence intervals (shaded area). Results of the overall Friedman’s rank test are provided.

Wet and dry seasons in Cambodia reflect the seasonal abundance of *Aedes* spp. We detected significant differences in anti-SGH, anti-AeD7L1, anti-AeD7L2, and anti-AeD7L1+2 IgG levels between wet and dry seasons for the same subset of 18 Cambodian children who were sampled at seven consecutive visits (**Figure 3**). Additionally, the seasonal fluctuation for AeD7L1 and the composite marker AeD7L1+2 was stronger (moderate effect size, Kendall’s W = 0.33 and 0.30, respectively) than that for SGH or AeD7L2 (small effect size, Kendall’s W = 0.17 and 0.19, respectively). This was further reflected in the Conover post-hoc test that highlighted significant differences between the medians of six visits for both AeD7L1 and AeD7L1+2 whereas only for three visits for SGH and AeD7L2 (**Supplementary Table 2**). No significant differences were observed for the Ab responses against BSA during the wet and dry seasons (**Supplementary Figure S3**).

### 3.4 Children who developed asymptomatic dengue had higher anti-AeD7L1+2 antibody titers at baseline than those who developed symptomatic dengue

**Figure 4:**
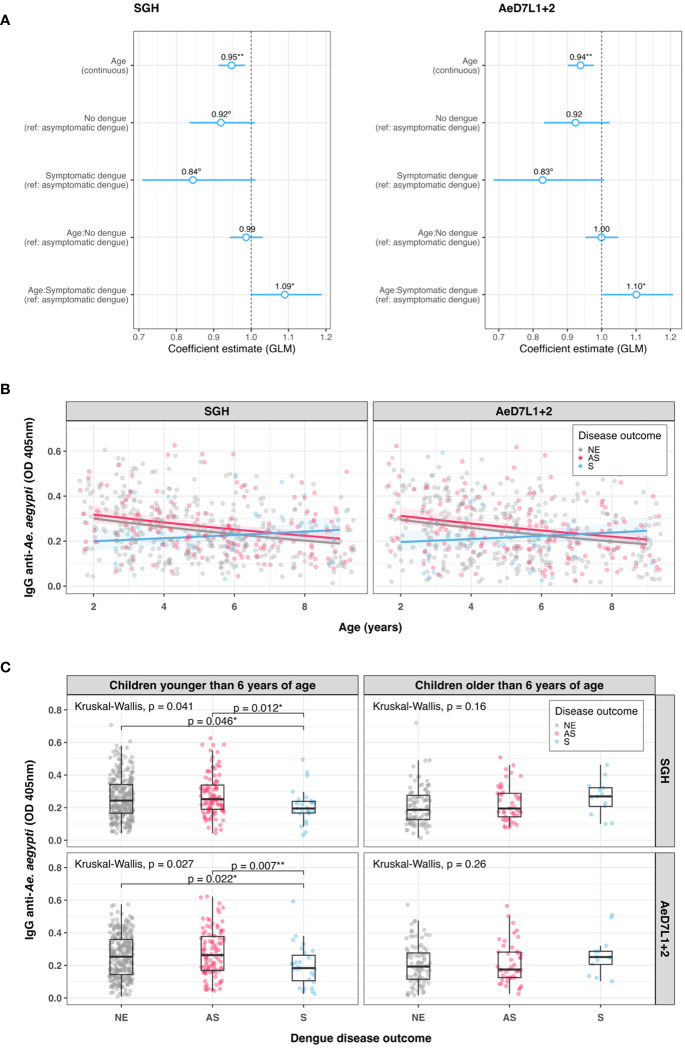
Trends in baseline anti-*Aedes aegypti* IgG responses. **(A)** Exponentiated regression coefficient estimates for the generalized linear model fitted to the IgG data per assay. The circles represent the exponentiated mean coefficient estimate (the value shown above the corresponding circle). The horizontal lines represent the 95% confidence intervals of the estimate. The referent group was “asymptomatic” for both disease outcomes and the age × disease outcome interaction term. **(B)** Scatterplot of standardized ELISA IgG optical density (OD) values with age at study baseline for both SGH and AeD7L1+2 (n = 563). The solid lines visualize the marginal effect of the age × disease outcome interaction from the generalized linear model (GLM) regression fitted to the antibody data. **(C)** Dot and box plots of IgG OD values for SGH and AeD7L1+2, comparing different dengue disease outcomes for children younger or older than 6 years. The solid black horizontal line within the box plots is the median; the lower and upper borders are, respectively, the first and third quartiles; the vertical bars indicate the minimum and maximum values. Results of the overall Kruskal–Wallis test and the post-hoc Dunn’s test for pairwise comparisons are provided. GLM, generalized linear model; OD, optical density; NE, no event; AS, asymptomatic dengue; S, symptomatic dengue; SGH, salivary gland homogenate. Significance levels **p < 0.01; *p < 0.05; °*p* < 0.1.

We tested serum samples of all children naïve at baseline (n = 563) for the response against AeD7L1+2 to assess associations with disease outcomes (i.e., no dengue infection, asymptomatic dengue, and symptomatic dengue). Correlation coefficients between *Ae. aegypti* SGH and the AeD7L1+2 recombinant proteins remained strong for the full cohort (r = 0.85, r_s_ = 0.86). To investigate associations with anti-*Aedes* Ab levels at baseline and disease, we first fitted Cox PH models that consider time to any dengue infection. We did not observe any significant differential risk for dengue between children with high or low baseline Ab levels for either SGH or AeD7L1+2 (**Supplementary Tables 3**, **4**). We then tested the distribution of baseline Ab responses to SGH and AeD7L1+2 with age and disease outcome groups. Accounting for age, disease outcome, and an age × disease outcome interaction term, multivariate models revealed that overall Ab responses decreased with increasing age for both SGH and AeD71+2 (**Figure 4A**). However, the Ab response of children who developed symptomatic dengue increased with age compared to children who developed asymptomatic dengue (test of age × disease outcome interaction term; **Figure 4A**). Marginal effect plots of the age × disease outcome interaction from the GLM regression indicate a cross-over in baseline Ab responses at 6 years old (**Figure 4B**). Indeed, when comparing median baseline Ab levels between disease outcomes, a significant difference existed among children younger than 6 years who developed asymptomatic *vs.* symptomatic dengue for both SGH and AeD7L1+2 (*p* = 0.046 and *p* = 0.022, respectively, **Figure 4C**). Children older than 6 years did not exhibit differences in baseline median Ab responses between disease outcome groups (**Figure 4C**).

### 3.5 Antibody reactivity to AeD7L1+2 recombinant salivary proteins is specific to *Aedes* mosquitoes

**Figure 5:**
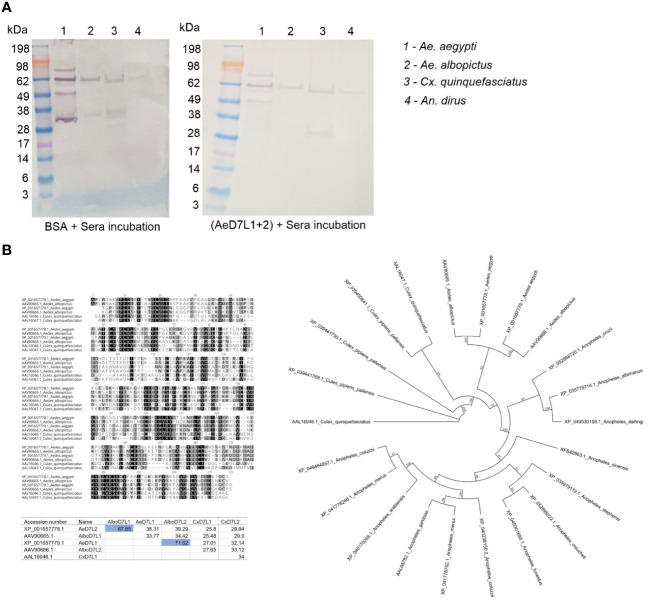
Specificity of AeD7L1+2 recombinant proteins to *Aedes* mosquitoes. **(A)** Western blotting showing reactivity of Cambodian sera to salivary gland homogenate (SGH) of *Aedes aegypti*, *Aedes albopictus*, *Culex quinquefasciatus*, and *Anopheles dirus* without (left) and with (right) pre-incubation with AeD7L1+2 recombinant proteins. **(B)** Multiple alignments of D7L proteins from *Ae. aegypti*, *Ae. albopictus*, and *Cx. quinquefasciatus* using MUSCLE. The matrix indicates the percentages of identities between D7L proteins of different species. Color shading indicates percentage identity between sequences, going from 100% (black) to 0% identity (white). The evolutionary history was inferred using the maximum likelihood method. Evolutionary analyses were conducted in Geneious using the Paup plugin.

Since Cambodians are exposed to the bites of several hematophagous insects during their life, we tested for potential cross-reactions between AeD7L1+2 proteins and salivary proteins from other mosquito species abundant in our study area using an inhibition immunoblot assay. We incubated SGH from *Ae. albopictus*, *Cx. quinquefasciatus*, and *An. dirus* with a pool of Cambodian sera reactive to *Ae. aegypti* SGH and observed recognition of several salivary proteins at diverse molecular weights, including the ones respective to AeD7L1+2 (**Figure 5A**, left panel). We then incubated the serum pool with AeD7L1+2 recombinant proteins to absorb AeD7L1+2-specific Abs. The serum reactivity at 28–38 kDa (corresponding to the MW of AeD7L1 and AeD7L2 proteins) was lost for both *Ae. aegypti* and *Ae. albopictus* SGH (**Figure 5A**, right panel). However, the sera remained reactive against a *Cx. quinquefasciatus* SGH protein of equivalent molecular weight, suggesting that Cambodian children have anti-*Culex* Abs that do not cross-react with AeD7L1+2. This further supports the notion that AeD7L1+2 recombinant proteins are specific to *Aedes* mosquitoes. Indeed, multiple pairwise alignments of the D7L proteins from *Ae. aegypti*, *Ae. albopictus*, and *Cx. quinquefasciatus* showed a high degree of divergence between species with few conserved areas of identity (**Figure 5B**, dark-shaded areas). In the *Aedes* genus, the AeD7L2 protein sequence is 67.85% identical to the AlboD7L1 sequence, and the AeD7L1 sequence is 71.62% identical to the AlboD7L2 sequence. Interestingly, AeD7L1 and AeD7L2 protein sequences are only 38.71% identical. Similarly, the phylogenetic analysis of the Culicidae confirmed that AeD7L1 clustered with AlboD7L2 whereas the AeD7L2 sequence clustered with AlboD7L1. Proteins from both *Aedes* species, however, were segregated from *Culex* D7L or *Anopheles* D7L proteins.

## 4 Discussion

*Ae. aegypti* SGH ELISAs are a useful tool to measure mosquito exposure (36) but are costly, laborious, and challenging to standardize between laboratories. Here, we propose an ELISA to measure Ab responses against recombinant *Ae. aegypti* D7L1+2 salivary proteins as a surrogate for monitoring human exposure to *Ae. aegypti* bites. Our hypothesis relies on measuring a rapid reduction of specific IgG Ab levels to AeD7L1+2 when exposure to *Ae. aegypti* is disrupted. *Ae. aegypti* abundance and dengue incidence are known to fluctuate throughout seasons, as has been evidenced by entomological indices and previous serosurveys (37, 38). As a proof of principle, the AeD7L1+2 assay uncovered seasonal weather patterns by capturing the waxing and waning of IgG Abs levels from dry to rainy seasons. This assay utilizes recombinant proteins, thereby forgoing reliance on insectary maintenance and manual salivary gland dissection and enabling standardization across different laboratories. It can be performed on a high-throughput and cost-effective platform and assessed with serum from a simple finger prick. The assay can be employed to identify hot spots with high biting rates (39) or to evaluate the effectiveness of vector control methods (40–42), which could then inform the allocation of scarce public health resources to areas with a high risk of *Aedes*-borne disease transmission. Additionally, having an accurate estimate of population exposure to *Aedes* mosquitoes will help raise community awareness and might encourage residents in high-risk areas to take protective measures (36).

The D7 family of salivary proteins is widely present across the order of bloodsucking Diptera (43) and likely regulates hemostasis by scavenging biogenic amines and leukotrienes, which are small molecules that play essential roles in inflammation, allergy, vascular permeability, and vascular tone (44). Indeed, salivary AeD7L knock-out mosquitoes required longer probing times than WT *Ae. aegypti*, confirming its importance in blood feeding (45). The D7L is known to be immunogenic in humans (46) and may serve as a potential exposure marker for *Anopheles gambiae* (47). Entomological parameters estimating *Ae. aegypti* densities are associated with SGH Ab responses, substantiating its use as a reference standard for the identification of new exposure markers (9). For the first time, this study formally correlates side-by-side paired responses to distinct *Ae. aegypti* salivary proteins to the whole *Ae. aegypti* SGH. Our unbiased screening approach provides the confidence that AeD7L1+2 assay is well suited to substitute *Ae. aegypti* SGH in ELISAs. The Abs to AeD7L1+2 seem to recognize both *Ae. aegypti* and *Ae. albopictus*, serving as an *Aedes* generic exposure tool with no detected cross-reactivity to *Cx. quinquefasciatus* or *An. dirus* mosquitoes SGH, a likely advantage to SGH assays, where the risk of cross-reactivity may be higher. These reactivity patterns are explained by the high sequence identity of D7L salivary proteins within the *Aedes* genus and their high degree of divergence compared to D7L salivary proteins from *Culex* or *Anopheles* species. Deploying this *Aedes* sp. tool can be advantageous for arboviral studies since both *Ae. albopictus* and *Ae. aegypti* effectively transmit many of the same viruses (48, 49). In certain occasions, however, specific information may be required to evaluate if the focus of transmission is more urban (*Ae. aegypti*) or rather in forested areas (*Ae. albopictus*). Our AeD7L1+2 assay could then be complemented with novel species-specific, yet less sensitive, specific salivary protein ELISAs.

Using our 3-year longitudinal pediatric cohort study (19), we show that IgG Ab levels against AeD7L1+2 are higher in naïve children younger than 6 years who developed asymptomatic (primary) dengue than those who developed symptomatic (primary) dengue. This observation may allude to a protective effect of AeD7L Abs in dengue disease outcomes. However, we were unable to replicate this association when including person-time at-risk in the analysis, highlighting the complexity of this “DENV–*Aedes* salivary proteins–anti-saliva Abs” triad. It has been shown that DENV-infected individuals have significantly higher Ab titers against total *Ae. aegypti* SGH than controls [3; 7; 12; 35]. Similarly, IgG levels against the Nterm-34 kDa and AgBR1 were higher in dengue patients without warning signs than in those with dengue with warning signs (50). However, Abs against the Nterm-34kDa peptide were not able to retrospectively identify individuals who developed DENV infection (14), and in Colombia, no statistically significant differences were found when comparing antibodies to D7L between febrile DENV-negative, non-febrile DENV-negative, and DENV-positive subjects (17), underscoring the uniqueness of each salivary protein and their distinct role in disease transmission. In the mosquito salivary glands, AeD7L expression is upregulated during DENV infection, and AeD7L is believed to bind DENV directly, forming protein complexes (51). Interestingly, AeD7L inhibited DENV infection in a human monocyte cell line and *in vivo* in a mouse model (51). The role of AeD7L1+2 Abs in infection adds further complexity to the DENV establishment in humans from endemic areas. AeD7L1+2 Abs may bind to D7 during transmission and help neutralize D7–DENV complexes, leading to a reduction in viral dissemination and primarily asymptomatic dengue. However, the neutralization capacity and the quality of AeD7L1+2 Abs from our dengue asymptomatic versus symptomatic subjects on D7–DENV complexes remain to be tested.

We acknowledge that more data are needed to validate the performance of the AeD7L1+2 assay in other populations especially in adults, given that our results are limited to Cambodian children. These validation studies should include longitudinal studies to assess *Ae. aegypti* seasonality and vector control interventions. Altogether, this will further instruct whether the combined AeD7L1+2 assay remains superior and/or the most generic (e.g., by detecting more Abs, including those against *Ae. albopictus*) or if the assay can be downsized to the D7L1 alone, given its high, yet lower, correlation with SGH and a similar ability to show the seasonal fluctuation in biting pressure. To expedite the universal application of the AeD7L1+2 assay, we are committed to openly sharing the AeD7L1+2 recombinant proteins with other research groups. The AeD7L1+2 proteins used in our assay are stable when kept refrigerated after a 4-month testing period, with only a minor decay at room temperature. This supports their simple deployment in resource-scarce settings, especially when developed into a quantitative lateral flow assay, which will in turn overcome the need for expensive equipment, such as an ELISA reader spectrophotometer, and inter-laboratory standardization. A limiting factor of our assay is the lack of a seropositivity cutoff, which is challenging to define given that most Cambodians are under constant *Ae. aegypti* biting pressure. Additionally, we did not study other Ab isotypes or IgG subclasses but focused on IgG responses given their abundance and robustness in freeze-and-thaw cycles (52–54). Future mechanistic studies would benefit from in-depth investigations into the humoral and cellular immune response to *Ae. aegypti* saliva to gain an understanding of how AeD7L1+2 Abs impact dengue disease outcome. Knowing the rate at which AeD7L Abs decay after disrupting mosquito–human contact will be critical to optimizing its use as an epidemiological tool. Abs against saliva of several vector species, including *Ae. aegypti*, are known to decline with age (11, 55–60). Nevertheless, how these dynamics impact dengue disease outcomes is not well understood. Discerning these age trends will be crucial when factoring them into the design of future studies. Overall, bridging these gaps will contribute to a more nuanced understanding of the interplay between immunity and *Aedes*-borne diseases and expose potential strategies for their prevention and control.

The highly sensitive and *Aedes*-specific novel AeD7L1+2 exposure assay will be useful in advancing the epidemiological understanding of *Aedes*-borne diseases. Our assay will encourage and facilitate the standardization of future serosurveys and epidemiological studies by its ability to provide a robust estimation of human–mosquito contact in a high-throughput fashion.

## Data availability statement

The original contributions presented in the study are included in the article/**Supplementary Material**. Further inquiries can be directed to the corresponding author.

## Ethics statement

The study protocol was approved by the institutional review boards (IRB) at the US National Institutes of Health and the National Ethics Committee on Human Research (NECHR) in Cambodia. The parents or guardians of all pediatric participants provided signed informed consent to participate in the study (ClinicalTrials.gov identifier: NCT03534245). Sera from healthy U.S. blood donors were collected following signed informed consent from participants in the National Institutes of Health Clinical Center IRB-approved protocol entitled Collection and Distribution of Blood Components from Healthy Donors for *In Vitro* Research Use (ClinicalTrials.gov identifier: NCT00001846). The studies were conducted in accordance with the local legislation and institutional requirements. Written informed consent for participation in this study was provided by the participants’ legal guardians/next of kin. The manuscript presents research on animals that do not require ethical approval for their study.

## Author contributions

SC: Conceptualization, Data curation, Formal analysis, Investigation, Project administration, Writing – original draft, Writing – review & editing. LW: Conceptualization, Data curation, Formal analysis, Methodology, Writing – original draft, Writing – review & editing. SN: Investigation, Methodology, Validation, Writing – review & editing. KT: Investigation, Writing – review & editing. PL: Investigation, Writing – review & editing, Formal analysis. JO: Investigation, Writing – review & editing. RS-C: Investigation, Writing – review & editing. AP: Investigation, Writing – review & editing. PV: Investigation, Writing – review & editing. DK: Investigation, Writing – review & editing. SL: Investigation, Writing – review & editing. RS: Investigation, Writing – review & editing. CL: Conceptualization, Data curation, Methodology, Project administration, Supervision, Writing – review & editing. RL: Project administration, Supervision, Writing – review & editing. RH: Project administration, Supervision, Writing – review & editing. CY: Conceptualization, Supervision, Writing – original draft, Writing – review & editing. JV: Project administration, Supervision, Writing – review & editing. EC: Resources, Writing – review & editing. JM: Formal analysis, Project administration, Supervision, Writing – original draft, Writing – review & editing. FO: Conceptualization, Formal analysis, Investigation, Methodology, Project administration, Supervision, Writing – original draft, Writing – review & editing.
