# Supplementary material for: Antibodies to Aedes aegypti D7L salivary proteins as a new serological tool to estimate human exposure to Aedes mosquitoes
Source: Front Immunol. 2024 May 1;15:1368066. doi: 10.3389/fimmu.2024.1368066 (PMC11094246; doi:10.3389/fimmu.2024.1368066)
Supplement: Supplementary file 1 [file DataSheet_1.docx]

Supplementary Material

**Antibodies to *Aedes aegypti* D7L salivary proteins as a new serological tool to estimate human exposure to *Aedes* mosquitoes.**

Sophana Chea^1†^, Laura Willen^2†^, Sreynik Nhek^1^, Piseth Ly^1^, Kristina Tang^2^, James Oristian^2^, Roberto Salas-Carrillo^2^, Aiyana Ponce^2^, Paola Carolina Valenzuela Leon^2^, Dara Kong^1^, Sokna Ly^2^, Ratanak Sath^2^, Chanthap Lon^1,2^, Rithea Leang^3,4^, Rekol Huy^3^, Christina Yek^1,2^, Jesus G. Valenzuela^2^, Eric Calvo^2^, Jessica E. Manning^1,2^, Fabiano Oliveira^1,2^*.

^1^ International Center of Excellence in Research, National Institute of Allergy and Infectious Diseases, Phnom Penh, Cambodia

^2^ Laboratory of Malaria and Vector Research, National Institute of Allergy and Infectious Diseases, National Institutes of Health, Bethesda, MD, USA

^3^ National Center for Parasitology, Entomology, and Malaria Control, Ministry of Health, Phnom Penh, Cambodia

^4^ National Dengue Control Program, Ministry of Health, Phnom Penh, Cambodia

Sophana Chea^1†^ and Laura Willen^2†^: These authors contributed equally to this work and share first authorship.

**Correspondence:** Fabiano Oliveira, loliveira@nih.gov

# Supplementary Figures

**Supplementary Figure 1.** **Western blots for reactivity of Cambodian sera with whole salivary gland homogenate of *Ae. aegypti*.** Each lane represents the serum reactivity of one Cambodian individual with SGH. MW: Molecular Weight; SGH: Salivary Gland Homogenate.

**Supplementary Figure 2. Ponceau staining and Western Blot of 18 recombinantly expressed *Ae. aegypti* salivary proteins.** Protein names and their estimated molecular weight are shown in the table. MW: Molecular Weight; SGH: Salivary Gland Homogenate.

**
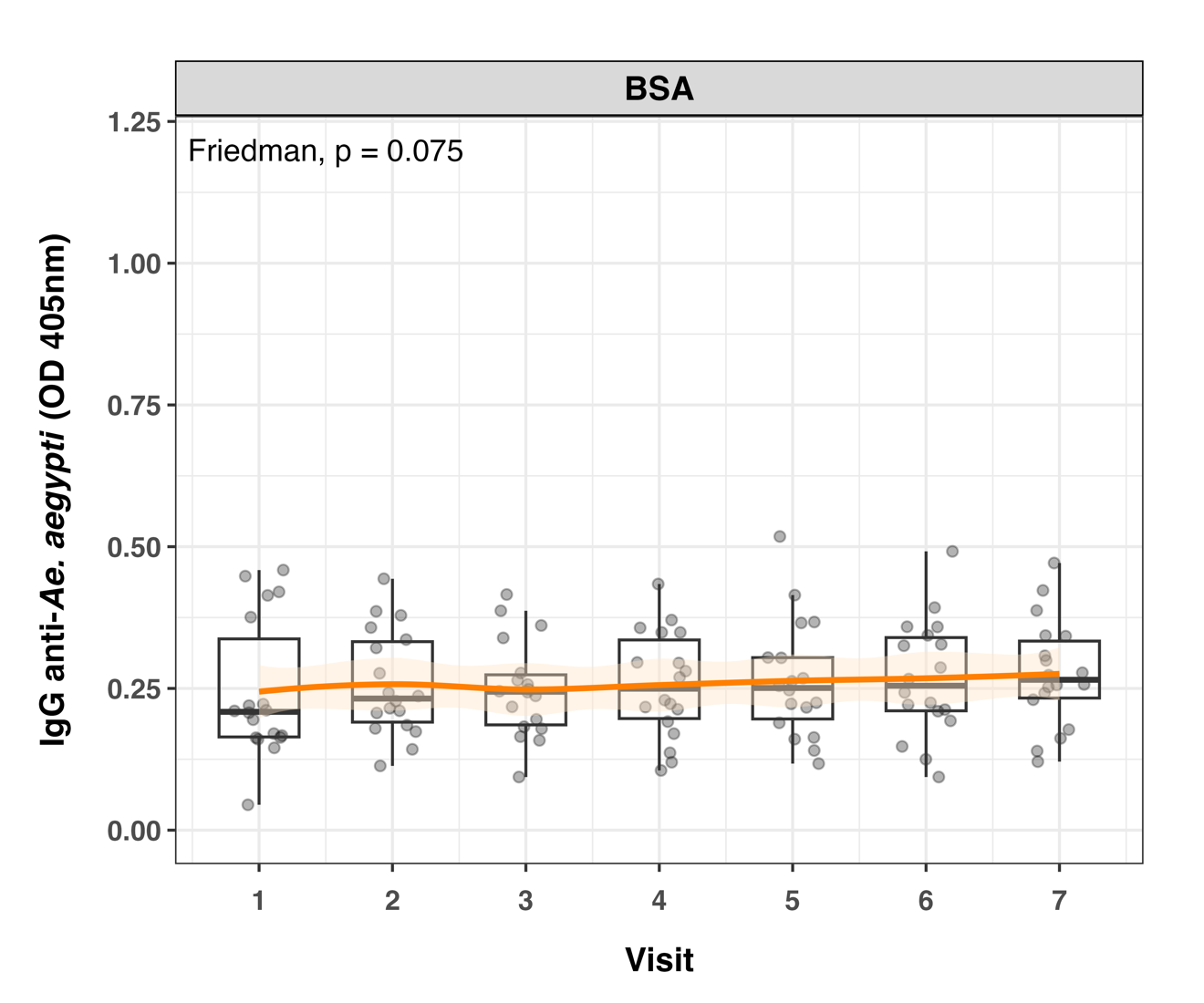
Supplementary Figure 4.** No significant differences were observed for the Ab responses against BSA during the wet and dry seasons
