# Supplementary material for: Antibodies to Aedes aegypti D7L salivary proteins as a new serological tool to estimate human exposure to Aedes mosquitoes
Source: Front Immunol. 2024 May 1;15:1368066. doi: 10.3389/fimmu.2024.1368066 (PMC11094246; doi:10.3389/fimmu.2024.1368066)
Supplement: Supplementary file 2 [file Table_1.docx]

Supplementary Material

**Antibodies to *Aedes aegypti* D7L salivary proteins as a new serological tool to estimate human exposure to *Aedes* mosquitoes.**

Sophana Chea^1†^, Laura Willen^2†^, Sreynik Nhek^1^, Piseth Ly^1^, Kristina Tang^2^, James Oristian^2^, Roberto Salas-Carrillo^2^, Aiyana Ponce^2^, Paola Carolina Valenzuela Leon^2^, Dara Kong^1^, Sokna Ly^2^, Ratanak Sath^2^, Chanthap Lon^1,2^, Rithea Leang^3,4^, Rekol Huy^3^, Christina Yek^1,2^, Jesus G. Valenzuela^2^, Eric Calvo^2^, Jessica E. Manning^1,2^, Fabiano Oliveira^1,2^*.

^1^ International Center of Excellence in Research, National Institute of Allergy and Infectious Diseases, Phnom Penh, Cambodia

^2^ Laboratory of Malaria and Vector Research, National Institute of Allergy and Infectious Diseases, National Institutes of Health, Bethesda, MD, USA

^3^ National Center for Parasitology, Entomology, and Malaria Control, Ministry of Health, Phnom Penh, Cambodia

^4^ National Dengue Control Program, Ministry of Health, Phnom Penh, Cambodia

Sophana Chea^1†^ and Laura Willen^2†^: These authors contributed equally to this work and share first authorship.

**Correspondence:** Fabiano Oliveira, loliveira@nih.gov

## Supplementary Tables

|  | Pearson Correlation | | | Spearman Correlation | | |
| --- | --- | --- | --- | --- | --- | --- |
| Recombinant protein | **Coefficient (r)** | ***p* value** | **0.95 CI** | **Coefficient (r_s_)** | ***p* value** | **0.95 CI** |
| AeD7L1+2 | 0.91 | 4.26e-049 | 0.87 – 0.94 | 0.92 | 4.96e-52 | 0.89 – 0.94 |
| AeD7L1 | 0.82 | 8.75e-32 | 0.75 – 0.87 | 0.84 | 1.00e-34 | 0.78 – 0.89 |
| AeD7L2 | 0.76 | 5.93e-25 | 0.67 – 0.83 | 0.82 | 1.70e-31 | 0.75 – 0.87 |
| AeApyrase + AeD7L1+2 + NIH-27 + NIH-23 | 0.81 | 8.24e-31 | 0.74 – 0.86 | 0.87 | 7.95e-41 | 0.83 – 0.91 |
| AeApyrase + AeD7L1+2 + NIH-27 | 0.84 | 2.12e-34 | 0.78 – 0.88 | 0.90 | 8.98e-48 | 0.87 – 0.93 |
| AeApyrase + AeD7L1+2 + NIH-23 | 0.79 | 1.46e-27 | 0.71 – 0.84 | 0.85 | 4.05e-37 | 0.80 – 0.90 |
| AeApyrase + NIH-23 + NIH-27 | 0.72 | 1.68e-21 | 0.625 – 0.7957 | 0.78 | 2.03e-27 | 0.71 – 0.84 |
| AeD7L1+2 + NIH-27 + NIH-23 | 0.90 | 1.20e-45 | 0.86 – 0.93 | 0.93 | 1.03e-56 | 0.91– 0.95 |
| AeD7L1+2 + NIH-27 | 0.90 | 3.73e-46 | 0.86 – 0.93 | 0.93 | 1.14e-56 | 0.91– 0.95 |
| BSA | 0.17 | 0.051 | -0.001 – 0.34 | 0.15 | 0.10 | -0.03 – 0.31 |

**Supplementary Table 1. Correlation coefficients, *p* values and confidence intervals for Pearson and Spearman correlation between SGH and all tested combinations of *Ae. aegypti* recombinant salivary proteins.** BSA: Bovine Serum Albumin; CI: Confidence Interval.

| SGH | | | | | | | D7L1+2 | | | | | |
| --- | --- | --- | --- | --- | --- | --- | --- | --- | --- | --- | --- | --- |
|  | **Visit 1** | **Visit 2** | **Visit 3** | **Visit 4** | **Visit 5** | **Visit 6** | **Visit 1** | **Visit 2** | **Visit 3** | **Visit 4** | **Visit 5** | **Visit 6** |
| Visit 2 | 0.052 |  |  |  |  |  | **0.01736** |  |  |  |  |  |
| Visit 3 | 0.742 | 0.102 |  |  |  |  | 0.55324 | **0.00278** |  |  |  |  |
| Visit 4 | 0.128 | 0.683 | 0.211 |  |  |  | 0.00576 | 0.68715 | **0.00083** |  |  |  |
| Visit 5 | 0.626 | 0.026 | 0.452 | **0.045** |  |  | 0.29625 | 0.00075 | 0.65348 | **0.00028** |  |  |
| Visit 6 | 0.029 | 0.683 | 0.045 | 0.452 | **0.012** |  | 0.00278 | 0.55324 | 0.00052 | 0.78819 | **0.00019** |  |
| Visit 7 | 0.452 | 0.245 | 0.626 | 0.452 | 0.211 | 0.128 | 0.19501 | 0.32265 | 0.05144 | 0.17631 | 0.01496 | 0.10885 |
| D7L1 | | | | | | | **D7L2** | | | | | |
|  | **Visit 1** | **Visit 2** | **Visit 3** | **Visit 4** | **Visit 5** | **Visit 6** | **Visit 1** | **Visit 2** | **Visit 3** | **Visit 4** | **Visit 5** | **Visit 6** |
| Visit 2 | **0.00038** |  |  |  |  |  | 0.2042 |  |  |  |  |  |
| Visit 3 | 0.44695 | **0.00558** |  |  |  |  | 0.7394 | 0.1119 |  |  |  |  |
| Visit 4 | 0.00032 | 0.92719 | **0.00472** |  |  |  | 0.0852 | 0.6829 | **0.0488** |  |  |  |
| Visit 5 | 0.68108 | 9.2e-05 | 0.20415 | **9.2e-05** |  |  | 0.3440 | 0.0256 | 0.4734 | **0.0109** |  |  |
| Visit 6 | 0.00029 | 0.92719 | 0.00403 | 0.92719 | **9.2e-05** |  | 0.0468 | 0.4734 | 0.0256 | 0.7114 | **0.0053** |  |
| Visit 7 | 0.06976 | 0.06976 | 0.35996 | 0.06577 | 0.02473 | 0.05761 | 0.4734 | 0.5600 | 0.3440 | 0.3440 | 0.0852 | 0.2042 |

**Supplementary Table 2.** ***P* value results of the** **post-hoc Conover -Iman test.** Differences in antibody responses between separate visits were tested with a Friedman rank test and a post-hoc Conover-Iman test. The *p* values highlighted in bold indicate significant (< 0.05 or lower) differences between two sequential visits. SGH: Salivary Gland Homogenate.

**Supplementary Table 3.** **Hazard ratios, confidence intervals and *p* values per risk factor tested for dengue seroconversion using Cox regression.** *Aedes aegypti* exposure was estimated using anti-SGH antibody levels. SGH: Salivary Gland Homogenate; HR: Hazard Ratio; CI: Confidence interval.

| **Risk factor** | **HR [CI]** | ***p*** |
| --- | --- | --- |
| ***Aedes aegypti* SGH salivary protein antibodies** (*ref: low*) |  |  |
| *High* | 1.3 [0.94-1.79] | 0.112 |
| **Sex** *(ref: female)* |  |  |
| *Male* | 1.04 [0.78-1.41] | 0.777 |
| **Age** (per year) | 1.08 [0.98-1.19] | 0.132 |
| **Educational status** *(ref: in school)* |  |  |
| *Not in school* | 0.83 [0.53-1.29] | 0.402 |
| **Socioeconomic class** *(ref: Lower/ very poor)* |  |  |
| *Middle/*u*pper* | 0.99 [0.67-1.45] | 0.950 |
| **No. of domestic water containers at home** | 0.10 [0.94-1.06] | 0.927 |
| **No. of toilets in the home** | 1.18 [0.97-1.42] | 0.094 |
| **Use of bednets** *(ref: rarely/never)* |  |  |
| *Regularly/ all of the time* | 0.89 [0.57-1.39] | 0.610 |
| **Use of insecticide spray** *(ref: does not use)* |  |  |
| *Uses insecticide spray* | 0.69 [0.50-0.94] | 0.020 |
| **Use of larvicide** *(ref: applies larvicide to water)* |  |  |
| *Does not apply larvicide to water* | 1.22 [0.81-1.83] | 0.349 |
| **How often mosquito coils burned** *(ref: daily/often)* |  | 0.023 |
| *Never* | 1.28 [0.91-1.79] | 0.154 |
| *Sometimes/rarely (1-3 times/wk)* | 0.75 [0.49-1.16] | 0.195 |

**Supplementary Table 4.** **Hazard ratios, confidence intervals and *p* values per risk factor tested for dengue seroconversion using Cox regression.** *Aedes aegypti* exposure was estimated using anti-D7L1+2 antibody levels. HR: Hazard Ratio; CI: Confidence interval.

| **Risk factor** | **HR [CI]** | ***p*** |
| --- | --- | --- |
| ***Aedes aegypti* D7L1+2 salivary protein antibodies** (*ref: low*) |  |  |
| *High* | 1.2 [1.4-0.9] | 0.341 |
| **Sex** *(ref: female)* |  |  |
| *Male* | 1 [2.4-0.8] | 0.872 |
| **Age** (per year) | 1.1 [1.2-1] | 0.157 |
| **Educational status** *(ref: in school)* |  |  |
| *Not in school* | 0.8 [1.5-0.5] | 0.391 |
| **Socioeconomic class** *(ref:* L*ower/ very poor)* |  |  |
| *Middle/*u*pper* | 1 [2.5-0.7] | 0.914 |
| **No. of domestic water containers at home** | 1 [2.6-0.9] | 0.972 |
| **No. of toilets in the home** | 1.2 [1.1-1] | 0.095 |
| **Use of bednets** *(ref: rarely/never)* |  |  |
| *Regularly/ all of the time* | 0.9 [1.9-0.6] | 0.617 |
| **Use of insecticide spray** *(ref: does not use)* |  |  |
| *Uses insecticide spray* | 0.7 [1-0.5] | 0.020 |
| **Use of larvicide** *(ref: applies larvicide to water)* |  |  |
| *Does not apply larvicide to water* | 1.2 [1.4-0.8] | 0.285 |
| **How often mosquito coils burned** *(ref: daily/often)* |  | 0.023 |
| *Never* | 1.3 [1.2-0.9] | 1.365 |
| *Sometimes/rarely (1-3 times/wk)* | 0.7 [1.2-0.5] | <0.0001 |
